# Kisspeptin-10 regulates glycosaminoglycan and decorin content in human cardiac fibroblast cultures

**DOI:** 10.1007/s43440-026-00870-6

**Published:** 2026-05-20

**Authors:** Paulina Radwańska, Maciej Olkiewicz, Lucyna Piera, Jacek Drobnik

**Affiliations:** Department of Pathophysiology, Chair of General and Experimental Pathology, Medical University of Lodz, Żeligowskiego 7/9, Łódź, 90-752 Poland

**Keywords:** Decorin, Glycosaminoglycan, Human cardiac fibroblasts, Kisspeptin-10

## Abstract

**Background:**

Cardiac fibroblasts play a key role in extracellular matrix (ECM) remodelling through the synthesis of glycosaminoglycans and proteoglycans (PGs), such as decorin; however, the effects of kisspeptin-10 (KiSS-10) on these components in the heart remain unknown. The aim of this study was to determine the influence of KiSS-10 on glycosaminoglycan and decorin content in human cardiac fibroblast cultures and identify its mechanism.

**Methods:**

The effects of KiSS-10 on glycosaminoglycans and decorin were assessed in human cardiac fibroblast cell line, with or without signalling inhibitor. Glycosaminoglycan levels were determined by the Farndale method, decorin secretion by enzyme-linked immunosorbent assay (ELISA), and decorin expression by quantitative polymerase chain reaction (qPCR).

**Results:**

KiSS-10 treatment caused a dose-dependent increase in glycosaminoglycan content in both cells and medium. This stimulatory effect was abolished by blocking G-protein–coupled receptor 54 (GPR54) with peptide 234 or focal adhesion kinase (FAK) with FAK inhibitor 14 (FAKi). Combining KiSS-10 with phospholipase C inhibitor (D609) did not alter the glycosaminoglycan level compared to KiSS-10 alone. KiSS-10 increased decorin secretion but not expression. Combining peptide 234 with KiSS-10 did not alter media decorin level compared to KiSS-10 alone.

**Conclusions:**

KiSS-10 significantly elevated glycosaminoglycan and decorin accumulation in human cardiac fibroblast cultures. This regulatory effect on glycosaminoglycan level appears to be dependent on GPR54 activation and FAK signalling, but not on phospholipase C function. Hence, KiSS-10 may be involved in ECM remodelling in the heart.

## Introduction

Most eukaryotic cells produce complex macromolecules that belong to the family of proteoglycans (PGs), which act as components of the pericellular and extracellular matrix (ECM). PGs consist of a protein core and at least one covalently-attached glycosaminoglycan chain [1]. Glycosaminoglycans are linear polysaccharides constructed of repeating disaccharide units, each containing an amino sugar, either N-acetylglucosamine or N-acetylgalactosamine, and a uronic acid, i.e., glucuronic acid or iduronic acid. Currently, six types of glycosaminoglycans have been identified in mammals: chondroitin sulfate, dermatan sulfate, keratan sulfate, heparan sulfate, heparin, and hyaluronic acid. All but hyaluronic acid are sulphated and attach to proteins to form PGs [2]. These ECM components are known to be involved in cardiac homeostasis regulation and disease pathogenesis.

All PGs are known to regulate collagen fibrillogenesis and maintain tissue integrity and matrix stiffness, and are able to influence mechanotransduction by binding to cell surface receptors. Changes in their structure can also influence the myocardium [1]. Indeed, some PGs play important roles in heart development and in preventing cardiovascular malformation [3]. However, PG accumulation within the heart is linked to pathological remodelling of the ECM and the development of cardiac fibrosis [4].

After synthesis, PGs are stored in secretory granules, inserted into the plasma membrane, or secreted into the ECM. This group includes the small leucine-rich PGs (SLRPs). One SLRP, decorin, consists of a single glycosaminoglycan chain of dermatan sulphate or chondroitin sulphate attached to a core protein [2]. Decorin has cardioprotective, anti-inflammatory, and anti-fibrotic properties. It is known to attenuate the transformation of fibroblasts into myofibroblasts and inhibit transforming growth factor-β (TGF-β) signalling [3, 5, 6].

Cardiac remodelling is also influenced by kisspeptin, whose production is controlled by the KiSS-1 metastasis-suppressor gene (KISS1). All functional kisspeptins have a biologically active 10-amino acid fragment at the C-terminus that binds to a receptor. These hormones can activate a wide variety of intracellular signalling pathways *via* G-protein coupled receptor 54 (GPR54), also known as KiSS1R, AXOR12, or hOT7T175 [7, 8]. By binding to this receptor, kisspeptin stimulates Gαq/11-coupled cascades involving phospholipase C (PLC) activation; PLC plays crucial roles in inositol 1,4,5-trisphosphate (IP3) release, intracellular Ca^2+^ mobilization, diacylglycerol (DAG) accumulation, and protein kinase C (PKC) activation.

Other secondary effectors of the KiSS-1/GPR54 signalling pathway include extracellular signal-regulated kinase 1 and 2 (ERK1/2), p38 mitogen-activated protein kinase (p38 MAPK), and phosphatidylinositol-3-kinase (PI3K)/protein kinase B (Akt) [9]. Moreover, in some types of cells, kisspeptin is able to phosphorylate focal adhesion kinase (FAK) through GPR54 [10–12]. While kisspeptin and GPR54 expression have been detected in the cardiovasculature, including the heart, aorta, coronary artery, and umbilical vein [13], their role in the cardiovascular system remains unclear.

A few reports indicate that the KiSS-1/GPR54 system can influence cardiac function and blood vessel properties. However, while kisspeptin may have an inotropic effect on the hearts of rats, mice, and humans in vitro [14], it has not been found to influence blood pressure or heart rate in healthy individuals in vivo [15]. It appears to be involved in umbilical vein and coronary artery vasoconstriction [16], and attenuates cutaneous peripheral blood flow, elevates permeability and plasma extravasation, and promotes the development of oedema in mice [17]. Kisspeptin-10 (KiSS-10) treatment was also reported to have pro-fibrotic effects in rat hearts manifested as increased numbers of collagen fibres in the cardiac muscle and pathological remodelling [18]. Furthermore, Dinh et al. [19]. note that blocking kisspeptin receptor activation aggravates uremic cardiomyopathy in rats, and that a high dose of peptide 234 hastens the development of uremic cardiomyopathy; the authors attribute this to fibrotic TGF-β pathway stimulation.

Our prior research indicates that KiSS-10 has profibrotic activity and elevates collagen content in human cardiac fibroblasts (in vitro) and mouse hearts (in vivo) [20]. This suggests that the hormone can also influence the production of other ECM components; however, the effect of KiSS-10 on glycosaminoglycan production currently remains unknown. As the fibrotic process in the myocardium can be modulated by decorin, a key PG involved in ECM organization, we hypothesized that KiSS-10 regulates glycosaminoglycan accumulation and decorin production in the heart. Therefore, the aim of the current study was to determine the role of KiSS-10 in the regulation of sulphated glycosaminoglycans and decorin level in human cardiac fibroblasts, and identify the mechanism by which this takes place.

## Materials and methods

### Reagent preparations

The following reagents were used: KiSS-10 (synthetic peptide; sequence: Tyr–Asn–Trp–Asn–Ser–Phe–Gly–Leu–Arg–Phe–NH_2_; catalog no. orb372481, Biorbyt, Cambridge, UK), peptide 234 (kisspeptin 234, kisspeptin receptor antagonist; peptide sequence: Ac-D-Ala-Asn-Trp-Asn-Gly-Phe-Gly-D-Trp-Arg-Phe-NH_2_; catalog no. 3881, Tocris, Ellisville, MO, USA), D609 (selective competitive phosphatidylcholine-specific phospholipase C (PC-PLC) inhibitor, *O*-(Octahydro-4,7-methano-1*H*-inden-5-yl) carbonopotassium dithioate; catalog no. 1437, Tocris, Ellisville, MO, USA), focal adhesion kinase inhibitor 14 (FAKi, 1,2,4,5-Benzenetetramine tetrahydrochloride; catalog no. 3414, Tocris, Ellisville, MO, USA).

The study employed certain reagents with specific properties. Firstly, peptide 234 is a selective competitive antagonist of the kisspeptin receptor, specifically blocking KiSS-10–mediated signalling. Also, FAKi was used to selectively inhibit FAK by preventing its autophosphorylation at Tyr397; the inhibitor demonstrates no significant activity against other kinases, including epidermal growth factor receptor (EGFR), platelet-derived growth factor receptor (PDGFR), and insulin-like growth factor 1 receptor (IGF1R). Another reagent was D609, a selective competitive inhibitor of PC-PLC, although it has also been reported to inhibit sphingomyelin synthase and suppress nitric oxide (NO) production induced by lipopolysaccharide (LPS) and interferon gamma (IFNγ) in certain cell types.

All of these reagents were initially dissolved in water and stored at -20 °C until further dilution in medium immediately before use. The reagents were prepared at the following concentrations: 1.0 × 10^− 11^ – 1.0 × 10^− 5^ mol/L (KiSS-10), 1.0 × 10^− 7^ mol/L (peptide 234), 1.0 × 10^− 6^ – 1.0 × 10^− 5^ mol/L (D609), and 1.0 × 10^− 7^- 1.0 × 10^− 6^ mol/L (FAKi).

### Cell culture

The in vitro study was conducted on an immortalized human cardiac fibroblast cell line (ABM, Richmond, BC, Canada) obtained from the ventricles of a healthy adult human heart. The cells were cultured in Dulbecco’s Modified Eagle’s Medium (DMEM) (Biowest, Nuaillé, France) with 10% (v/v) foetal bovine serum (FBS) (Biowest, Nuaillé, France), gentamicin (25 µg/mL) (Gibco, Thermo Fisher Scientific, Waltham, Massachusetts, USA), amphotericin B (0.25 µg/mL) (Capricorn Scientific, Ebsdorfergrund, Germany), insulin (5 µg/mL) (Thermo Fisher Scientific, Waltham, Massachusetts, USA), and vitamin C (50 µg/mL) (Sigma-Aldrich, Saint Louis, Missouri, USA). The cells were cultured on plates coated with collagen (10 µg/cm^2^) (Sigma-Aldrich, Saint Louis, Missouri, USA) at 37 °C under a humidified atmosphere of 5% CO_2_. The cells were grown to confluence before being trypsynized (Trypsin–EDTA 1X in PBS, Biowest, Nuaillé, France) and passaged. All experiments were carried out between passages six to eleven.

The human cardiac fibroblasts were incubated in DMEM containing 3% (v/v) bovine serum, insulin, vitamin C and antibiotics at the concentrations given above. In addition, depending on the experimental variant, this was supplemented with 10^− 11^ – 10^− 5^ mol/L of KiSS-10, peptide 234 alone (10^− 7^ mol/L), or peptide 234 (10^− 7^ mol/L) with KiSS-10 (10^− 8^ mol/L), FAKi alone (10^− 7^ − 10^− 6^ mol/L), FAKi (10^− 7^ − 10^− 6^ mol/L) with KiSS-10 (10^− 8^ mol/L), D609 alone (10^− 6^ -10^− 5^ mol/L) or D609 (10^− 6^ -10^− 5^ mol/L) with KiSS-10 (10^− 8^ mol/l). All incubations were performed for 96 h.

The medium was changed every day. On Day 5 of the experiment, the cells were stained with trypan blue (Sigma-Aldrich, Saint Louis, Missouri, USA) and total fibroblast and necrotic cell numbers were counted in a Bürker chamber. Samples of media were collected each day and assayed for glycosaminoglycan content, as were fibroblasts collected on the final day. Decorin content was measured in the media samples collected on Day 5. Decorin expression in the human cardiac fibroblasts was also assessed at 24 and 96 h.

### Glycosaminoglycan content assay

Total sulfated glycosaminoglycan content was measured using a modified Farndale method, and a 1,9-dimethylmethylene blue assay (DMMB). Briefly, the samples were dried at 60 °C, then incubated for one hour in a water bath at 73 °C with a solution of 0.75 M NaOH (Stanlab, Lublin, Poland) and 25 mM natrium borate (Merck, Darmstadt, Germany). The samples were neutralized with 6 M HCl (Stanlab, Lublin, Poland) to approximately pH 7. Following this, 7.2 µL of 100% TCA (POCH S.A., Gliwice, Poland) was added to precipitate proteins. The samples were then centrifuged at 6000 rpm for 30 min, and the supernatant was dissolved in 6 mL of absolute ethanol (POCH S.A., Gliwice, Poland). To precipitate glycosaminoglycans, the samples were stored overnight in a refrigerator at -20 °C, and then centrifuged at 12 500 rpm for 30 min. The precipitate with glycosaminoglycans was collected and dissolved in distilled water. Each sample (50 µL) was added to 1.2 mL of DMMB-reagent, consisting of 51 mM DMMB (Sigma-Aldrich, USA), 45 mM glycine (Sigma-Aldrich, USA), and 41 mM NaCl (POCH S.A., Gliwice, Poland). The pH of the solution was adjusted to 3.0 with 1 M HCl. The absorbance of each sample was evaluated at λ = 525 nm on a spectrophotometer and compared to the standard curve [21, 22].

### Human decorin enzyme-linked immunosorbent assay (ELISA)

The concentrations of decorin within the human cardiac fibroblast media were determined using a Human Decorin ELISA kit (catalog no. QY-E03148, Qayee Biotechnology, Co., Ltd., Shanghai, China) (detection range: 0.5–100 ng/mL, species specificity: human, no-cross reaction with analogues). The ELISA assays were carried out according to the manufacturer’s instructions. Absorbance was measured with an Epoch Microplate Spectrophotometer plate reader (BioTek Instruments Inc, USA) at 450 nm. The secretion was expressed as the concentrations released into the culture medium by a specific number of cells.

### Isolation of total RNA

Total RNA was extracted from fibroblast cell culture using a Total RNA Mini Test (A&A Biotechnology, Gdynia, Poland) according to the manufacturer’s protocol. RNA quality and quantity were determined using a NanoDrop Spectrophotometer (Thermo Fisher Scientific, Waltham, Massachusetts, USA). After 96 h, the fibroblasts were lysed directly in the culture wells by adding fenozol (800 µL/1 × 10^6^ cells). After 5-min incubation at 50 °C, each lysate was supplemented with chloroform (200 µl/1 × 10^6^ cells), gently mixed, incubated for 3 min at room temperature, and centrifuged at 12 000 x g for 10 min. The upper phase containing RNA was collected and supplemented with isopropanol (250 µL/1 × 10^6^ cells). The mixture was mixed, transferred to minicolumns and centrifuged at 12 000 rpm for 1 min. Each sample was then washed with wash solution and centrifuged at 12 000 rpm for 1–2 min. This step was repeated three times. The purified total RNA was eluted by nuclease-free water. Samples of total RNA were stored at -20 °C until further analysis.

### Reverse transcription

Reverse transcription was performed using a PrimeScriptTM RT reagent Kit (Perfect Real Time) (Takara Bio Inc., Kusatsu, Shiga, Japan) according to the manufacturer’s protocol. A mixture consisting of total RNA (500 ng), primer solution (Oligo dT Primer, 50 µM, 0.5 µL; Random 6-mers, 100 µM, 2 µL), reverse transcriptase solution with RNase inhibitor (PrimeScript RT Enzyme Mix, 0.5 µL), reaction buffer containing nucleotides (5x PrimeScript Buffer, 2 µL) and nuclease-free water (to 10 µL) was prepared on ice. Following this, the whole sample was incubated for 15 min at 42 °C. Finally, the reaction was stopped by heating the mixture for 10 s at 85 °C. The reactions were carried out using a C1000TM Thermal Cycler (Bio-Rad Laboratories, Hercules, California, USA). The cDNA obtained was stored at -20 °C until further analysis.

### Quantitative polymerase chain reaction (qPCR)

The expression of decorin in the fibroblast cells was determined by quantitative PCR. Briefly, 2µL of cDNA was added to the following reaction mixture (18 µL): 5X Colorless GoTaq^®^Flexi Buffer, 4 mM MgCl_2_, PCR nucleotide Mix (0.2 mM), GoTaq^®^G2 Hot Start Polymerase (1.25 u/µL) (Promega GmbH, USA catalog no: M7406); EvaGreen Fluorescent DNA Stain (ABO, Gdansk, Poland) and 0.5 mM of each primer. The DNA polymerase was activated and the cDNA denatured for 10 min at 95 °C. The samples were then subjected to 55 cycles consisting of 95 °C for 10 s, 60 °C for 30 s and 72 °C for 1 s. The reaction mixture was then cooled at 40 °C for 30s.

Ribosomal protein lateral stalk subunit P0 (RPLP0), tyrosine 3-monooxygenase/tryptophan 5-monooxygenase activation protein zeta (YWHAZ) and glyceraldehyde-3-phosphate dehydrogenase (GAPDH) were used as a reference gene. The reference genes were assessed using the following sets of primers: human *RPLP0* forward 5′-GGCACCATTGAAATCCTGAG-3′ and reverse 5′-GAAGGGGGAGATGTTGAGC-3′ (Roche, Indianapolis, IN, USA); human YWHAZ forward 5′-AAGTGCAATGGAGACCTTGG-3′ and reverse 5′-GTTGCCCTAGATGCAGAAGG-3′ (Roche, Indianapolis, IN, USA); human *GAPDH* forward 5’-TCGGAGTCAACGGATTTG-3’ and reverse 5’-CAACAATATCCACTTTACCAGAG-3’ (Roche, Indianapolis, IN, USA); human decorin forward 5’-TTCACGCATTGATTCTTGTC-3’ and reverse 5’- GCTGATTCTTGGACAGATAAAG-3’ (Sigma-Aldrich, Saint Louis, Missouri, USA).

Nuclease-free water (Promega, Madison, WI, USA) was used as a negative control. Gene expression of untreated human cardiac fibroblasts was used as a calibrator. Each reaction was conducted in duplicate. LightCycler^®^ 96 software (Roche, Indianapolis, USA) was used to determine the relative gene expression.

### Statistical analysis

The normality of data was checked using the Shapiro-Wilk test. The homogeneity of variance was estimated using Levene’s test. Variables were classified as parametric if they demonstrated a normal distribution (Shapiro-Wilk test, *p* > 0.05) and homogeneity of variance (Levene’s test, *p* > 0.05). Variables that did not meet these assumptions were treated as nonparametric. Descriptive statistics were recorded as means and standard deviations (mean ± SD) for parametric data, and as medians with interquartile ranges (median [IQR]) for nonparametric data. Comparisons between pairs of groups were assessed by the two-tailed independent t-test (parametric data) or Mann-Whitney U-test (nonparametric data). For comparisons involving multiple groups, the data did not meet the assumptions for parametric ANOVA; in such cases, the data was analysed using the Kruskal-Wallis test, followed by multiple pairwise comparisons of mean ranks for all groups. Differences were considered as significant at *p* < 0.05. All analyses were performed using Statistica 13.1 PL (Statsoft, Poland).

## Results

### The effect of KiSS-10 on glycosaminoglycan content in human cardiac fibroblasts and culture media

**Fig. 1:**
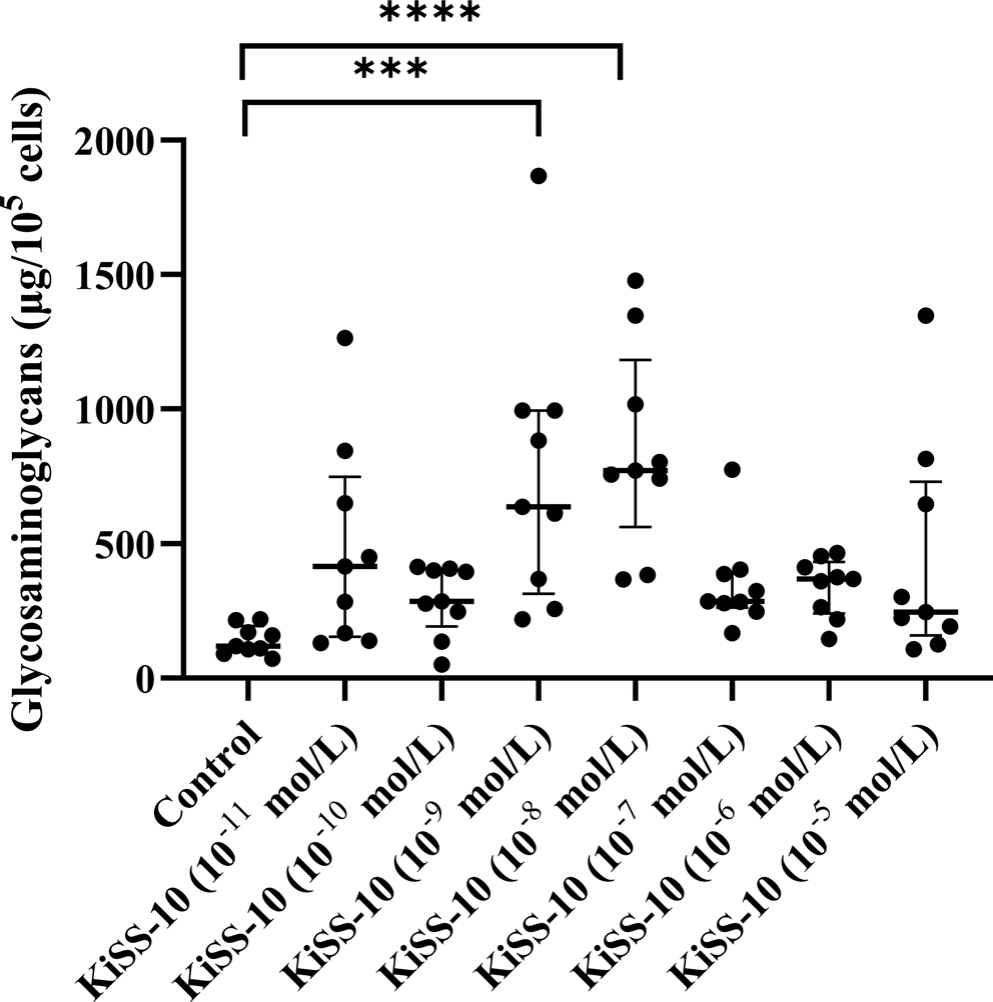
The influence of KiSS-10 (10^− 11^ − 10^− 5^ mol/L) on glycosaminoglycan content in human cardiac fibroblasts and culture media. Individual data points represent single observations (biological replicates), *n* = 9. The horizontal lines indicate the median, and the error bars represent the IQR. Significant differences were identified by the Kruskal-Wallis test, followed by multiple pairwise comparisons of mean ranks for all groups; the differences are indicated by asterisks (***p < 0.001, ****p < 0.0001 – significant difference compared to the control). Abbreviations: IQR – interquartile range; KiSS-10 – kisspeptin-10

The Kruskal–Wallis test demonstrated a significant overall effect of KiSS-10 treatment on glycosaminoglycan content in human cardiac fibroblasts and culture media (H = 29.17, N_1−8_ = 9, *p* = 0.0001). Following a significant Kruskal-Wallis test, multiple pairwise comparisons of mean ranks for all groups indicated that treatment with KiSS-10 at 10⁻⁹ mol/L (*p* = 0.00095) and 10⁻⁸ mol/L (*p* = 0.000035) resulted in significantly higher glycosaminoglycan levels compared to controls. No significant changes in glycosaminoglycan levels were observed following treatment with 10^− 11^ mol/L (*p* = 0.0904), 10^− 10^ mol/L (*p* = 0.9585), 10^− 7^ mol/L (*p* = 0.3531), 10^− 6^ mol/L (*p* = 0.2433), or 10^− 5^ mol/L KiSS-10 (*p* = 0.5781) (Fig. 1).

### The impact of KiSS-10 and the combined treatment with KiSS-10 and peptide 234 (kisspeptin receptor antagonist) on glycosaminoglycan content in human cardiac fibroblasts and culture media

**Fig. 2:**
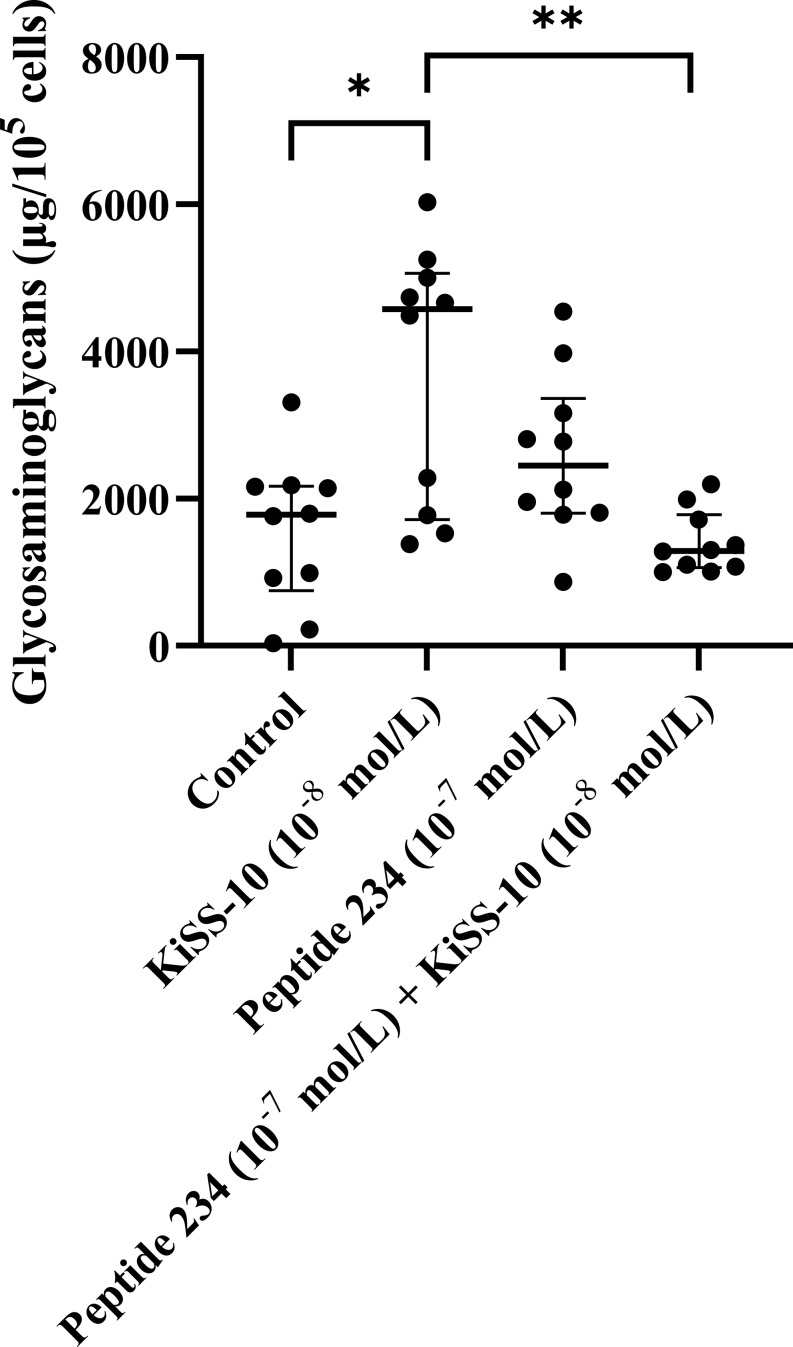
Changes in the glycosaminoglycan content in human cardiac fibroblasts and culture media under the influence of KiSS-10 alone (10^− 8^ mol/L) or with peptide 234 (10^− 7^ mol/L). Individual data points represent single observations (biological replicates), *n* = 10. The horizontal lines indicate the median, and the error bars represent the IQR. Significant differences were identified by the Kruskal-Wallis test, followed by multiple pairwise comparisons of mean ranks for all groups; the differences are indicated by asterisks (*p < 0.05, **p < 0.01 – significant difference between the groups). Abbreviations: IQR – interquartile range; KiSS-10 – kisspeptin-10; peptide 234 – kisspeptin receptor antagonist

The Kruskal-Wallis test indicated a significant overall effect of the experimental treatment on glycosaminoglycan content in human cardiac fibroblasts and culture media (H = 13.47, N_1-4_ = 10, *p* = 0.0037). Multiple pairwise comparisons of mean ranks for all groups following a significant Kruskal–Wallis test revealed that the fibroblasts treated with 10⁻⁸ mol/L of KiSS-10 demonstrated significantly (*p* = 0.0396) higher glycosaminoglycan content compared to untreated controls. Those receiving 10⁻⁸ mol/L of KiSS-10 with 10⁻⁷ mol/L peptide 234 demonstrated significantly lower glycosaminoglycan compared to 10⁻⁸ mol/L KiSS-10 alone (*p* = 0.0074). No differences in glycosaminoglycan level were found between the culture treated with peptide 234 (10^− 7^ mol/L) and untreated controls (*p* = 0.5322), or the combination of peptide 234 (10^− 7^ mol/L) with KiSS-10 (10^− 8^ mol/L) and controls (*p* = 1.0000) (Fig. 2).

### The impact of KiSS-10 and the combined treatment with KiSS-10 and phospholipase C inhibitor (D609) on glycosaminoglycan content in human cardiac fibroblasts and culture media

**Fig. 3:**
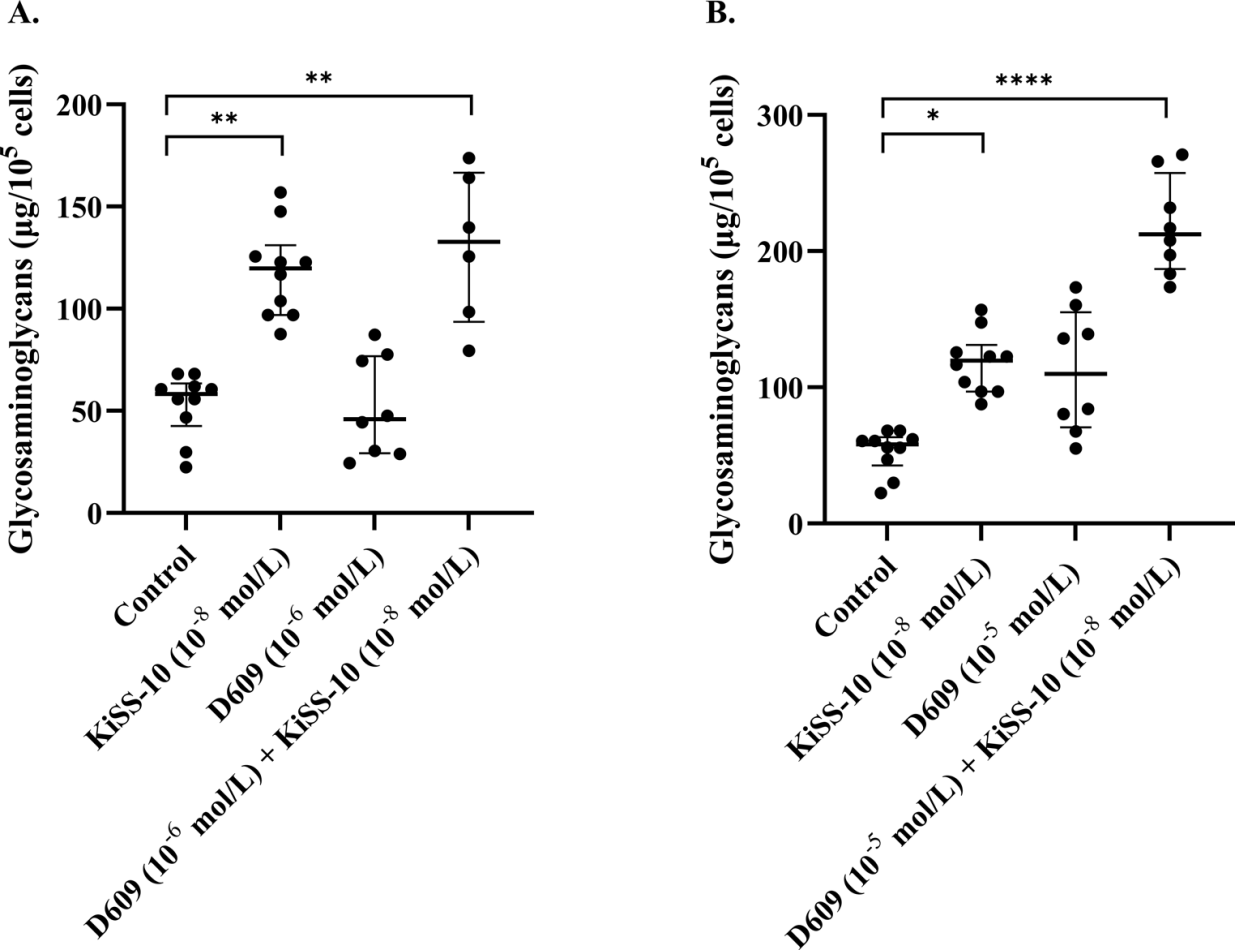
The impact of KiSS-10 and combined KiSS-10 with D609 on glycosaminoglycan content. **(A)** Cells treated with KiSS-10 (10⁻⁸ mol/L) alone or in combination with D609 (10⁻⁶ mol/L). **(B)** Cells treated with KiSS-10 (10⁻⁸ mol/L) alone or in combination with D609 (10⁻⁵ mol/L). Individual data points represent single observations (biological replicates: *n* = 3–5). Each biological replicate was assayed in duplicate. The horizontal lines indicate the median, and the error bars represent the IQR. Significant differences were identified by the Kruskal-Wallis test, followed by multiple pairwise comparisons of mean ranks for all groups; the differences are indicated by asterisks (*p < 0.05, **p < 0.01, ****p < 0.0001 – significant difference between the groups) Abbreviations: D609 - phospholipase C inhibitor; IQR – interquartile range; KiSS-10 – kisspeptin-10

In the experiment with 10^− 6^ mol/L D609, the Kruskal–Wallis test indicated a significant overall effect of the experimental conditions on glycosaminoglycan content in human cardiac fibroblasts and culture media (H = 24.50, N_1_ = 10, N_2_ = 10, N_3_ = 8, N_4_ = 6, *p* < 0.0001). Human cardiac fibroblasts treated with 10^− 8^ mol/L KiSS-10 demonstrated markedly higher glycosaminoglycan levels compared to untreated cells (*p* = 0.0018), as determined by multiple pairwise comparisons of mean ranks for all groups. No significant change in glycosaminoglycan accumulation was observed between cells treated with KiSS-10 combined with 10⁻⁶ mol/L D609 and cells treated with KiSS-10 alone (*p* = 1.0000). Combined treatment with KiSS-10 (10^− 8^ mol/L) and D609 (10^− 6^ mol/L) resulted in significantly higher level of glycosaminoglycan compared to controls (*p* = 0.0027). No significant differences in glycosaminoglycan accumulation were observed between cells exposed to 10^− 6^ mol/L D609 compared to controls (*p* = 1.0000) (Fig. 3A).

In the experiment with 10⁻⁵ mol/L D609, the Kruskal–Wallis test also revealed a significant overall effect of treatment on glycosaminoglycan content in human cardiac fibroblasts and culture media (H = 27.53, N_1_ = 10, N_2_ = 10, N_3_ = 8, N_4_ = 8, *p* < 0.0001). Multiple pairwise comparisons of mean ranks showed that fibroblasts treated with 10⁻⁸ mol/L KiSS-10 had significantly higher glycosaminoglycan levels than untreated controls (*p* = 0.0250), whereas no significant differences were observed between KiSS-10 (10^− 8^ mol/L) and KiSS-10 combined with 10^⁻5^ mol/L D609 (*p* = 0.0702). Combined treatment with KiSS-10 (10^− 8^ mol/L) and D609 (10^− 5^ mol/L) resulted in a significant elevation of glycosaminoglycan content compared to controls (*p* = 0.000001). No significant changes in glycosaminoglycan accumulation were found between D609 alone and controls (*p* = 0.1300) (Fig. 3B).

### The effect of KiSS-10 and the combined treatment with KiSS-10 and FAK inhibitor 14 (FAKi) on glycosaminoglycan levels in human cardiac fibroblasts and culture media

**Fig. 4:**
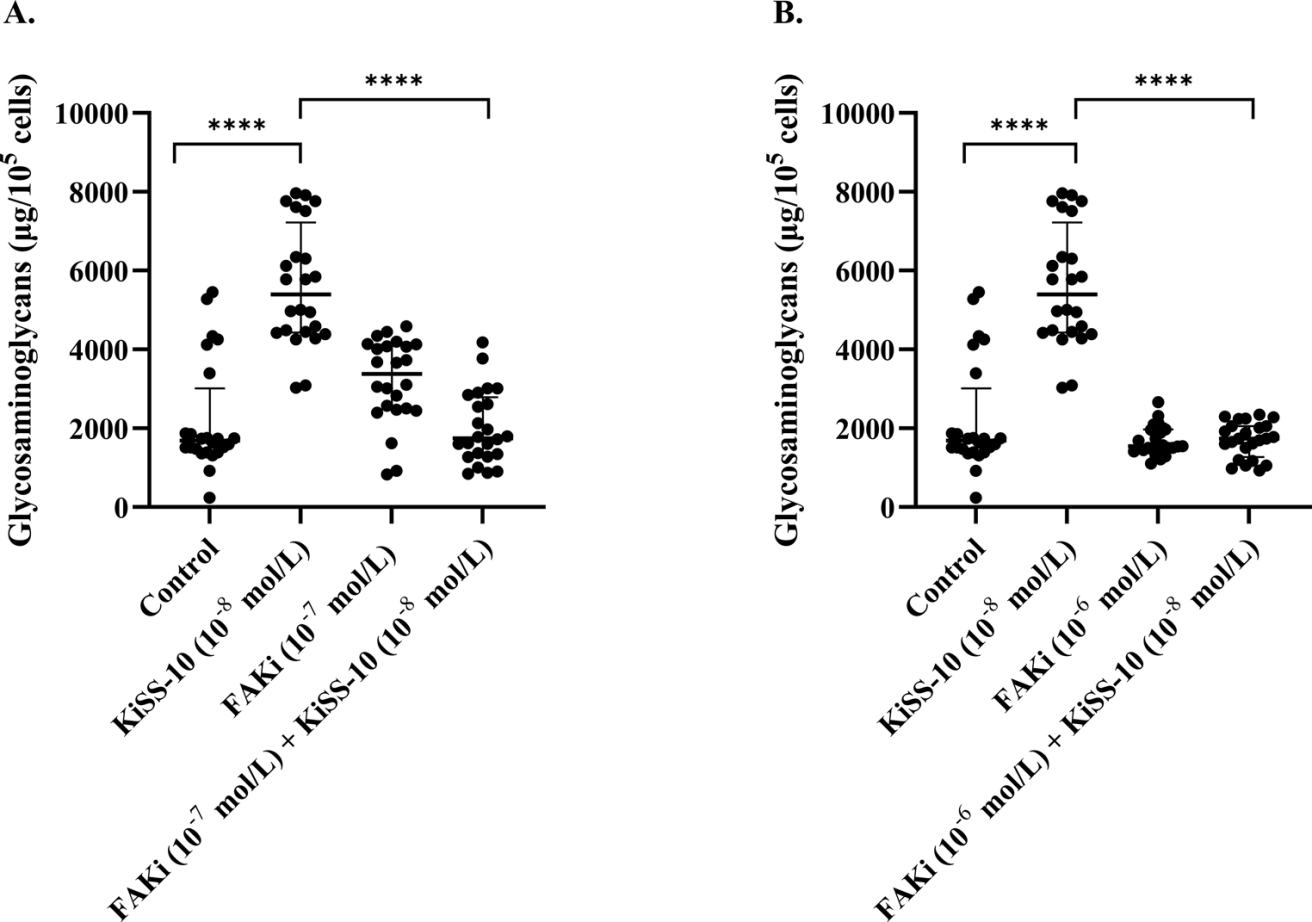
The effect of KiSS-10 and combined KiSS-10 with FAKi on glycosaminoglycan levels. **(A)** Cells treated with KiSS-10 (10⁻⁸ mol/L) alone or in combination with FAKi (10^− 7^ mol/L) **(B)** Cells treated with KiSS-10 (10⁻⁸ mol/L) alone or in combination with FAKi (10^− 6^ mol/L) Individual data points represent single observations (biological replicates: *n* = 12). Each biological replicate was assayed in duplicate The horizontal lines indicate the median, and the error bars represent the IQR. Significant differences were identified by the Kruskal-Wallis test, followed by multiple pairwise comparisons of mean ranks for all groups; the differences are indicated by asterisks (****p < 0.0001 – significant difference between the groups). Abbreviations: FAKi – focal adhesion kinase inhibitor 14; IQR – interquartile range; KiSS-10 – kisspeptin-10

In the experiment with 10⁻⁷ mol/L FAKi, the Kruskal-Wallis test indicated a significant overall effect of treatment on glycosaminoglycan content in human cardiac fibroblasts and culture media (H = 52.01, N_1−4_ = 24, *p* < 0.0001). Multiple pairwise comparisons of mean ranks following a significant Kruskal–Wallis test revealed that treatment with 10^− 8^ mol/L of KiSS-10 resulted in a significant increase of glycosaminoglycan content compared to controls (*p* < 0.000001). In contrast, combined treatment with KiSS-10 (10^− 8^ mol/L) and FAKi (10⁻⁷ FAKi) did not differ from controls (*p* = 1.0000) and resulted in a significant reduction of glycosaminoglycan content compared to KiSS-10 alone (*p* < 0.000001). No significant changes in glycosaminoglycan accumulation were found between FAKi treatments (10⁻⁷ mol/L) and controls (*p* = 0.1954) (Fig. 4A).

In the 10⁻⁶ mol/L FAKi experiment, the Kruskal–Wallis test indicated a significant overall effect of the experimental conditions on glycosaminoglycan content in human cardiac fibroblasts and culture media (H = 49.77, N_1−4_ = 24, *p* < 0.0001). Multiple pairwise comparisons of mean ranks revealed that fibroblasts treated with KiSS-10 (10^− 8^ mol/L) alone exhibited significantly higher glycosaminoglycan levels than controls (*p* < 0.000001). By contrast, combined treatment with KiSS-10 (10^− 8^ mol/L) and FAKi (10⁻⁶ mol/L) did not differ from controls (*p* = 1.0000) and significantly decreased glycosaminoglycan content compared to KiSS-10 (10^− 8^ mol/L) alone (*p* < 0.000001). No significant differences were observed between FAKi (10⁻⁶ mol/L) alone and untreated cells (*p* = 1.0000) (Fig. 4B).

### The effect of KiSS-10 on decorin secretion and decorin expression in human cardiac fibroblast cultures

**Fig. 5:**
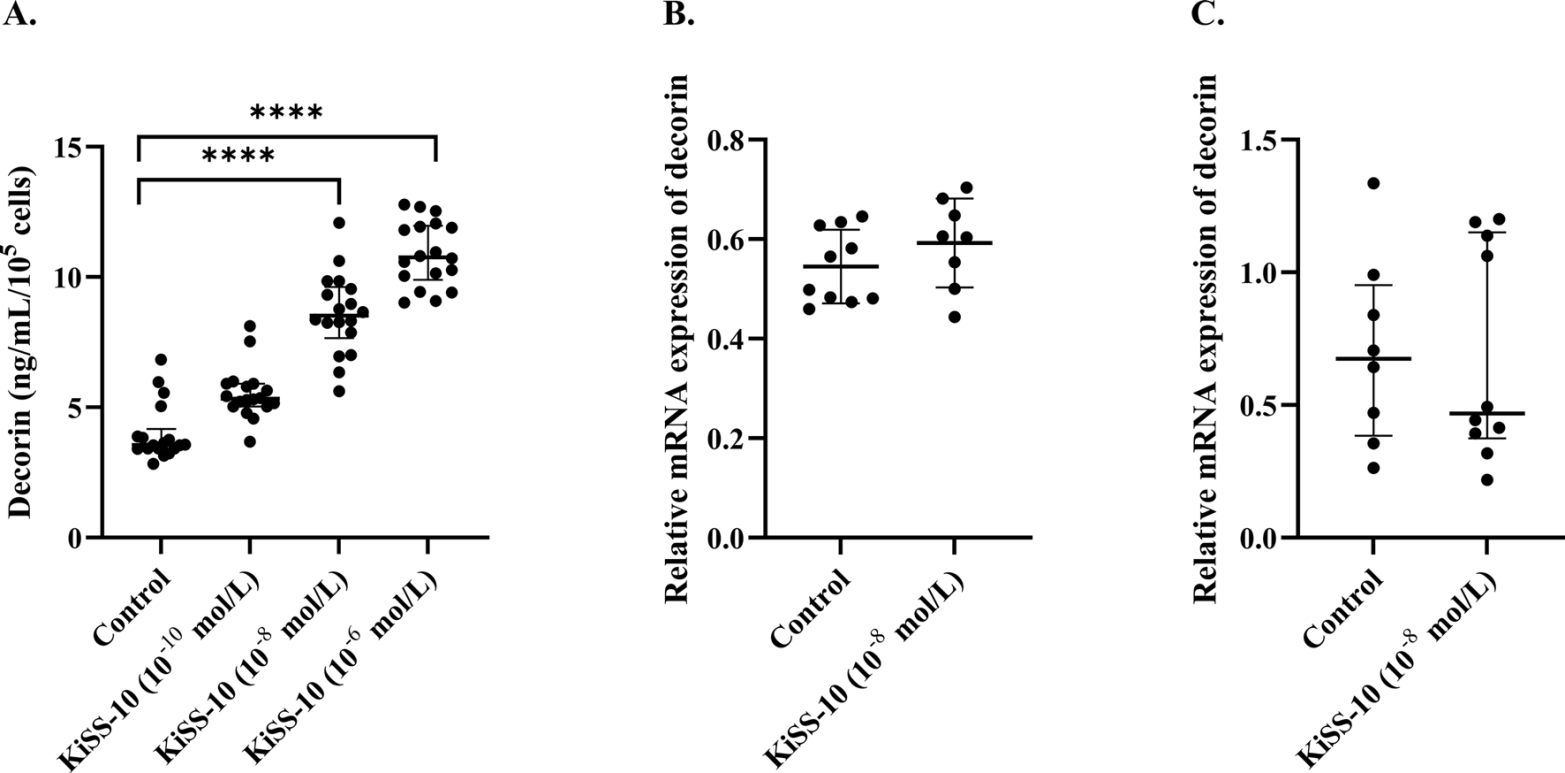
The effect of KiSS-10 on decorin secretion and expression in human cardiac fibroblast cultures. **(A)** Decorin secretion from human cardiac fibroblasts under the influence of KiSS-10. Individual data points represent single observations (biological replicates: *n* = 9). Each biological replicate was assayed in duplicate. The horizontal lines indicate the median, and the error bars represent the IQR. Significant differences were identified by the Kruskal-Wallis test, followed by multiple pairwise comparisons of mean ranks for all groups; the differences are indicated by asterisks (****p < 0.0001 – significant difference compared to the control). **(B)** Changes in the expression of decorin in human cardiac fibroblasts after 24 h of treatment with KiSS-10 (10^− 8^ mol/L) and in untreated controls. Individual data points represent single observations (biological replicates: *n* = 4–5). Each biological replicate was assayed in duplicate. The horizontal lines indicate the mean, and the error bars represent SD. Data were analysed with the two-tailed independent t-test. (**C**) Changes in the expression of decorin in human cardiac fibroblasts after 96 h of treatment with KiSS-10 (10^− 8^ mol/L) and in untreated controls. Individual data points represent single observations (biological replicates: *n* = 4–5). Each biological replicate was assayed in duplicate. The horizontal lines indicate the median, and the error bars represent the IQR. Data were analysed with the Mann-Whitney U-test. Abbreviations: IQR – interquartile range; KiSS-10 – kisspeptin-10; SD – standard deviation

The Kruskal–Wallis test demonstrated a significant overall effect of KiSS-10 treatment on decorin secretion by human cardiac fibroblasts (H = 58.53, N_1−4_ = 18, *p* < 0.0001). Following a significant Kruskal-Wallis test, multiple pairwise comparisons of mean ranks for all groups indicated that treatment with 10^− 8^ mol/L KiSS-10 or 10^− 6^ mol/L KiSS-10 significantly increased the level of decorin in the culture media compared to controls (*p* = 0.000006, *p* < 0.000001, respectively). No significant stimulatory effect was noted for the lowest KiSS-10 (10^− 10^ mol/L) concentration compared to non-treated cells (*p* = 0.3677) (Fig. 5A).

No significant changes in decorin expression were observed under the influence of 10^− 8^ mol/L KiSS-10 compared to controls. At 24 h, data were analysed using a two-tailed independent t-test (t₁₆ = −1.23, *p* = 0.2351), and at 96 h using the Mann-Whitney U-test (U = 38, N₁ = 8, N₂ = 10, *p* = 0.8968) (Fig. 5B and C).

### The impact of KiSS-10 and KiSS-10 with peptide 234 (kisspeptin receptor antagonist) on decorin content in culture media of human cardiac fibroblasts

**Fig. 6:**
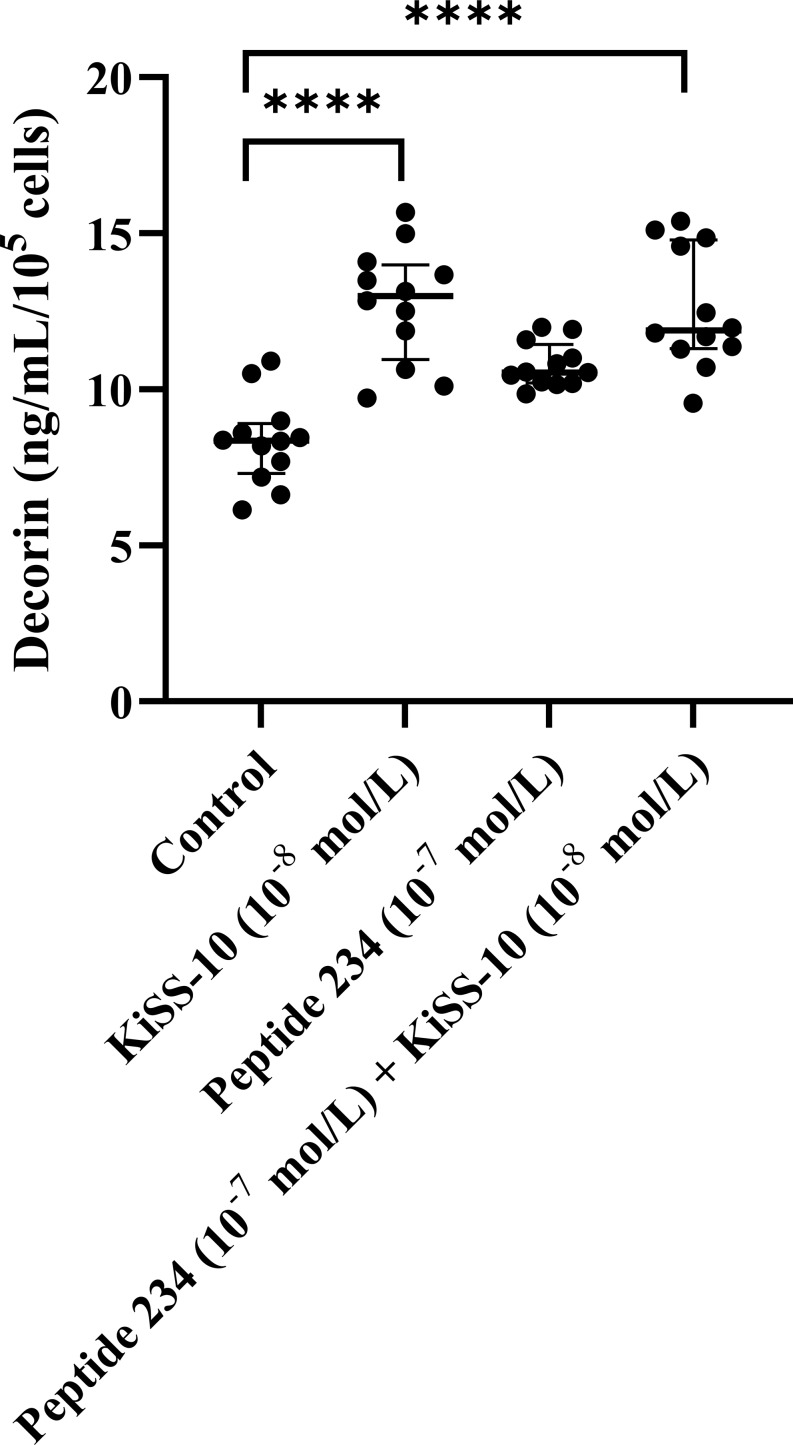
The content of decorin within human cardiac fibroblast culture media treated with KiSS-10 (10^− 8^ mol/L), peptide 234 (10^− 7^ mol/L), or peptide 234 (10^− 7^ mol/L) with KiSS-10 (10^− 8^ mol/L). Individual data points represent single observations (biological replicates), *n* = 12. The horizontal lines indicate the median, and the error bars represent the IQR. Significant differences were identified by the Kruskal-Wallis test, followed by multiple pairwise comparisons of mean ranks for all groups; the differences are indicated by asterisks (****p < 0.0001 – significant difference between the groups). Abbreviations: IQR – interquartile range; KiSS-10 – kisspeptin-10; peptide 234 – kisspeptin receptor antagonist

The Kruskal-Wallis test indicated a significant overall effect of treatment on glycosaminoglycan content in human cardiac fibroblasts and culture media (H = 26.98, N_1−4_ = 12, *p* < 0.0001). KiSS-10 (10^− 8^ mol/L) treatment resulted in significantly higher decorin secretion compared to untreated controls (*p* = 0.000035), as determined by multiple pairwise comparisons of mean ranks for all groups. However, no significant difference in media decorin level was found between treatment with KiSS-10 (10^− 8^ mol/L) and peptide 234 (10^− 7^ mol/L) and treatment with KiSS-10 alone (*p* = 1.0000). Combined treatment with KiSS-10 and peptide 234 resulted in significantly higher decorin secretion compared to controls (*p* = 0.00007). No significant difference in decorin level was noted between treatment with peptide 234 and untreated controls (*p* = 0.0858) (Fig. 6).

## Discussion

KiSS-10 increases glycosaminoglycan content in a dose-dependent manner in human cardiac fibroblasts and culture media. This effect is abolished by blocking GPR54 and by inhibition of FAK. In contrast, inhibition of PLC does not alter the KiSS-10-induced increase in glycosaminoglycan levels. Moreover, KiSS-10 enhances decorin core protein secretion by human cardiac fibroblasts without affecting its gene expression. This stimulatory effect of KiSS-10 on decorin levels in fibroblast culture medium is not abolished by kisspeptin receptor antagonist.

Treatment with KiSS-10 at concentrations of 10^− 9^ to 10^− 8^ mol/L was found to increase the glycosaminoglycan content in human cardiac fibroblasts and culture media. The greatest stimulation was observed under the influence of 10^− 8^ mol/L KiSS-10, where fibroblasts exhibited a 6.5-fold increase in glycosaminoglycan levels compared to untreated cells. The effect of KiSS-10 on glycosaminoglycan levels appears to be dose-dependent, exhibiting a non-linear response. Low concentrations (10⁻¹¹ and 10⁻¹⁰ mol/L) may be insufficient to effectively activate GPR54 receptors and trigger a cellular response. In contrast, higher concentrations (10⁻⁶ and 10⁻⁵ mol/L) did not increase glycosaminoglycan content, which may result from receptor desensitization, downregulation, or saturation [23–26]. Such regulatory processes are well-documented for G-protein coupled receptors and likely underlie the bell-shaped dose–response observed for many peptide ligands [27]. It should be also noted, that physiological concentrations of KiSS-10 in cardiac tissue are currently unknown. Circulating plasma levels have been reported to range from approximately 1.2 × 10⁻¹¹ mol/L in healthy adults [28, 29] up to ~ 1.4 × 10⁻⁸ mol/L in women during the third trimester of pregnancy [30]. These data indicate that the highest in vitro KiSS-10 concentrations (10⁻⁶ and 10⁻⁵ mol/L) exceed physiological plasma levels, but tissue-specific concentrations remain to be determined.

In addition, KiSS-10 has been found to elevate the levels of other ECM components, such as collagen, in human cardiac fibroblasts in vitro, as well as in mouse hearts in vivo [20]. This may suggest that treatment has a profibrotic effect, manifested as increased deposition of glycosaminoglycans and collagen in cardiac fibroblasts. This is consistent with Zhang et al. [18], who report that seven-day treatment with KiSS-10 induced cardiac fibrosis, characterized by increased collagen fibre deposition, impaired myocardial contractions, and fractured mitochondrial cristae in rat hearts.

However, the effect of kisspeptin on fibrotic processes depends on the target organ. Studies on mice have found kisspeptin-13 to inhibit pulmonary fibrosis caused by bleomycin; the mechanism is believed to be a complex one involving suppressing the inflammatory response and preventing the deposition of collagen and α-smooth muscle actin. Kisspeptin-13 also downregulates the expression of transforming growth factor-β (*Tgfb*), tumor necrosis factor-α (*Tnf*), collagen type I alpha 1 chain (*Col1a1*), actin α2 (*Acta2*), and matrix metalloproteinase-2 (*Mmp2*) [31]. However, there are currently no available data regarding the effects of kisspeptin-13 on glycosaminoglycan and decorin levels in cardiac fibroblasts.

Peptide 234 is a potent neutral antagonist of the GPR54 receptor that directly competes with KiSS-10 at its binding site, and exhibits high binding affinity, specificity and efficacy. Previous in vivo and in vitro studies have found peptide 234 administration to neutralise the effects of kisspeptin [32, 33]. Similarly, in the present study, treatment with 10^− 7^ mol/L peptide 234 negated KiSS-10 stimulation of glycosaminoglycan accumulation. Exposure of the fibroblasts to combined peptide 234 and KiSS-10 treatment resulted in a significant reduction in glycosaminoglycan content compared to KiSS-10 alone. This confirms that KiSS-10 has a direct effect on glycosaminoglycan content in human cardiac fibroblasts and is dependent on GPR54 receptor activity. It also suggests that peptide 234 may reduce the fibrotic effects of KiSS-10 by antagonizing kisspeptin receptor activity.

In contrast, Dinh et al. [19] report that peptide 234 exacerbated uremic cardiomyopathy in a rat model of chronic kidney disease (CKD), likely through activation of fibrotic TGF-β mediated pathways. The rats treated with peptide 234 demonstrated a significant increase in interstitial collagen content, along with overexpression of the fibrosis-related genes (*Tgfb*, collagen type III alpha 1 chain (*Col3a1*), connective tissue growth factor (*Ctgf*)) and cardiac remodelling markers (matrix metalloproteinase-9, *Mmp9*), compared to the untreated CKD controls. Lei et al. [31] found peptide 234 to abolish the antifibrotic effects of kisspeptin-13 in a mouse model of bleomycin-induced pulmonary injury and fibrosis.

Kisspeptin can activate a wide range of signalling pathways via the GPR54 receptor. The primary signalling mechanism involves typical Gαq/11-coupled cascades, including PLC activation, leading to the accumulation of IP3 and DAG; IP3 in turn promotes the mobilization of intracellular Ca^2+^, while DAG activates PKC. This pathway has been well documented as a route for kisspeptin signalling in gonadotropin-releasing hormone neurons [34] and in various cancer cell types [35]. There is also evidence that the PLC pathway plays a role in profibrotic processes in cardiac fibroblasts [36, 37]. Lee et al. [38] found PLC signalling to mediate the effects of fibroblast growth factor 23 (FGF-23) on cardiac fibroblast activity. More specifically, FGF-23 activates the PLC/IP3 signalling pathway, leading to the upregulation of Orai1 and/or TRPC1-mediated Ca^2+^ entry, which in turn, increases the proliferation and migration of human atrial fibroblasts.

Grabner et al. [39] propose that PLC may play a role in the development of cardiac myocyte hypertrophy in the left ventricle, with FGF-23 activating the phospholipase Cγ/calcineurin/nuclear factor activated T cells (NFAT) pathway through fibroblast growth factor receptor 4 (FGFR4). However, our present findings indicate that treatment with KiSS-10 (10^− 8^ mol/L) combined with the D609 did not result in any change in glycosaminoglycan level compared to cells treated with KiSS-10 alone. This suggests that the influence of KiSS-10 on glycosaminoglycan content is not modulated by PLC activity. However, it has been reported that GPR54 activation by kisspeptins can also induce additional signaling cascades, including the ERK1/2, p38 MAPK, and PI3K/Akt pathways [9]. These pathways could potentially play a role in the regulation of glycosaminoglycan levels in the heart. Nevertheless, further studies are required to clarify the specific contributions of these signaling mechanisms.

FAK signalling has been implicated in fibrogenesis across multiple organs, including the heart [40–42]. Rajshanker et al. [43] reported that FAK activity not only enhances collagen remodelling by tractional forces, but also inhibits collagen degradation by metalloproteinases in mouse embryonic fibroblasts. Similarly, Lagares et al. [40] indicate that FAK expression and activity are upregulated in fibroblast foci and remodelled vessels in lung fibrosis patients, and that inhibition of FAK, by pharmacological methods and small interfering RNA-mediated silencing, attenuates bleomycin-induced lung fibrosis in a mouse model. In contrast, in a study of FAK-deficient mice, Weng et al. [44] found the absence of FAK in liver epithelial cells to be associated with exacerbated liver injury and fibrosis compared to controls; this effect was linked to the activation of profibrotic pathways, including the hedgehog/smoothened signalling pathway.

In some organs, kisspeptin can also act through the FAK pathway. Wu et al. [12] demonstrated that in endometrial cancer cells, kisspeptin-regulated cell motility is influenced by FAK phosphorylation and Src-dependent activation of matrix metalloproteinase-2 (MMP-2). Also, Roseweir et al. [11] found KiSS-10 to activate FAK in extravillous trophoblast-derived cells and contribute to the inhibition of placental trophoblast cell migration. However, studies on human decidual stromal cells report that FAK phosphorylation was decreased by a kisspeptin agonist, but increased by a kisspeptin antagonist [45].

KiSS-10 has been found to stimulate phosphorylation of FAK at Tyr397 in human cardiac fibroblasts compared to unstimulated controls; it also appears to regulate collagen accumulation via FAK activity. Treatment with both KiSS-10 and FAKi resulted in lower intracellular collagen content compared to KiSS-10 treatment alone [20].

Since the FAK pathway mediates the effect of KiSS-10 on collagen deposition, another aim of the present study was to determine whether KiSS-10 treatment influences glycosaminoglycan levels via the FAK pathway. The fibroblasts and culture media receiving KiSS-10 plus FAKi (10^− 7^ or 10^− 6^ M mol/L) demonstrated significantly lower glycosaminoglycan content than those treated with KiSS-10 alone. This suggests that the stimulatory effect of KiSS-10 on glycosaminoglycan level was negated by inhibition of FAK autophosphorylation at Tyr397 [46, 47], and that the regulatory effect of KiSS-10 on glycosaminoglycan levels is dependent on FAK activity.

Another key regulator of fibrillogenesis and fibrosis is decorin. It is believed to play a role in reverse cardiac remodelling by directly inhibiting the TGF-β signalling pathway, and to reduce collagen production in human cardiac fibroblasts following TGF-β stimulation in vitro [48]. In contrast, Luxán et al. [49] report that decorin induces a pro-inflammatory environment in the heart microvasculature and may serve as an indicator of cardiac aging; decorin treatment also induces cardiomiocyte hypertrophy, increases the expression of interleukin 1β in endothelial cells, and compromises endothelial barrier function.

In the heart, decorin expression is upregulated by fasting [50], right ventricular pressure overload [51] and some hormones. Serum decorin concentrations are also increased by growth hormone, with a greater effect observed in men [52]. In addition, parathyroid hormone 2 receptor (PTH2R) activation induces decorin expression, which promotes wound healing [53]. Decorin expression was also found to be upregulated by progesterone in human immortalized epithelial cell lines derived from an ovarian endometrioma [54]. However, no studies to date have indicated a relationship between kisspeptin and decorin.

The present study is the first to demonstrate that KiSS-10 has an effect on decorin core protein levels in human cardiac fibroblasts; more specifically, its findings confirm that KiSS-10 stimulates the secretion of decorin from fibroblasts. Treatment with KiSS-10 (10^− 8^ mol/L and 10^− 6^ mol/L) significantly increased decorin protein levels in the culture media compared to untreated controls; however, it did not result in any significant change in decorin mRNA expression. This suggests that KiSS-10 may promote decorin accumulation in human cardiac fibroblasts by enhancing its biosynthesis at the post-transcriptional level. Indeed, various environmental and physiological factors are known to have a post-transcriptional influence on gene expression, which enables cells to adapt to changing conditions [55]. Nevertheless, the molecular mechanisms underlying the post-transcriptional regulation of decorin synthesis in response to KiSS-10 require further investigation.

Our findings also indicate that the stimulatory influence of KiSS-10 on decorin accumulation is not mediated by GPR54 receptor activation. Supplementation with peptide 234 (10^− 7^ mol/L) to cultures treated with KiSS-10 (10^− 8^ mol/L) did not alter decorin level in the medium compared to treatment with KiSS-10 alone. Other evidence suggests that kisspeptin may act through a GPR54-dispensable mechanism in the nervous system. KiSS-10 has been found to alleviate α-synuclein-mediated mitochondrial apoptosis in SH-SY5Y-derived neurons (a human neuroblastoma cell line), independently of kisspeptin receptors [56]. Moreover, studies conducted in pancreatic β-cells indicate that supraphysiological concentrations of kisspeptin can stimulate insulin secretion even in the absence of functional GPR54, suggesting that kisspeptin may act through alternative receptors [57]. However, due to the lack of data regarding the mechanism of action of kisspeptin on decorin production currently available in the literature, it is not possible to compare our present findings with those of previous studies.

## Conclusions

KiSS-10 application was found to significantly elevate glycosaminoglycan and decorin accumulation within human cardiac fibroblast culture, suggesting that it can regulate the deposition of certain ECM components in vitro. Interestingly, while such KiSS-10-driven glycosaminoglycan augmentation is dependent on GPR54 receptor activation and appears to be mediated by FAK, it is independent of PLC function. KiSS-10 was also found to regulate decorin deposition, but independently of the kisspeptin receptor; this process may hence take place through a GPR54-dispensable mechanism. It therefore appears that KiSS-10 may be involved in cardiac ECM remodelling. Approaches aimed at modulating kisspeptin levels may represent valuable therapeutic goals as these would support the regulation of ECM homeostasis and maintain myocardial tissue structure.

## Data Availability

The data will be made available from the corresponding author on reasonable request.

## Abbreviations

Acta2: Actin α2 gene
Akt: Protein kinase B
CKD: Chronic kidney disease
Col1a1: Collagen type I alpha 1 chain gene
Col3a1: Collagen type III alpha 1 chain gene
Ctgf: Connective tissue growth factor gene
DAG: Diacylglycerol
DMEM: Dulbecco’s Modified Eagle’s Medium
DMMB: 1,9-dimethylmethylene blue
ECM: Extracellular matrix
EGFR: Epidermal growth factor receptor
ELISA: Enzyme-linked immunosorbent assay
ERK1/2: Extracellular signal-regulated kinase 1 and 2
FAK: Focal adhesion kinase
FAKi: Focal adhesion kinase inhibitor 14
FBS: Foetal bovine serum
FGF-23: Fibroblast growth factor 23
FGFR4: Fibroblast growth factor receptor 4
GAPDH: Glyceraldehyde-3-phosphate dehydrogenase
GPR54: G-protein coupled receptor 54
IFNγ: Interferon gamma
IGF1R: Insulin-like growth factor 1 receptor
IP3: Inositol 1,4,5-trisphosphate
IQR: Interquartile range
KISS1: KiSS-1 metastasis-suppressor gene
KiSS-10: Kisspeptin-10
LPS: Lipopolysaccharide
MMP-2: Matrix metalloproteinase-2
Mmp2: Matrix metalloproteinase-2 gene
Mmp9: Matrix metalloproteinase-9 gene
NFAT: Nuclear factor of activated T cells
NO: Nitric oxide
p38 MAPK: p38 mitogen-activated protein kinase
PC-PLC: Phosphatidylcholine-specific phospholipase C
PDGFR: Platelet-derived growth factor receptor
PGs: Proteoglycans
PI3K: Phosphatidylinositol-3-kinase
PKC: Protein kinase C
PLC: Phospholipase C
PTH2R: Parathyroid hormone 2 receptor
qPCR: Quantitative polymerase chain reaction
RPLP0: Ribosomal protein lateral stalk subunit P0
SD: Standard deviation
SLRPs: Small leucine-rich proteoglycans
Tgfb: Transforming growth factor-β gene
TGF-β: Transforming growth factor-β
Tnf: Tumor necrosis factor-α gene
YWHAZ: Tyrosine 3-monooxygenase/tryptophan 5-monooxygenase activation protein zeta
